# Supplementary material for: Deletion of wfs1 Impairs Oligodendrocyte Precursor Cells Dorsal Distribution and Myelination Through the wfs1-hmgcs1 Axis in Zebrafish
Source: Biology (Basel). 2026 Aug 16;15(16):1402. doi: 10.3390/biology15161402 (PMC13510151; doi:10.3390/biology15161402)
Supplement: Supplementary file 1 [file biology-15-01402-s001.zip › File S1-Transcriptome sequencing data and differentially expressed genes (DEGs) in wfs1-- zebrafish at 5 dpf/2.KEGG/wfs1KOvsWT/DOWN/src/dre04012.html]

dre04012


- dre:544664

- Down regulated genes

544664(-5.14896748950436)

- dre:544664

- Down regulated genes

544664(-5.14896748950436)

- dre:571431

- Down regulated genes

571431(-6.42862549702529)

- dre:571431

- Down regulated genes

571431(-6.42862549702529)

- dre:58100

- Down regulated genes

58100\_1(-4.1286149197908)

- dre:544664

- Down regulated genes

544664(-5.14896748950436)

- dre:497384

- Down regulated genes

497384\_1(-2.05267237809407)

- dre:100000252

- Down regulated genes

100000252\_1(-1.7718817168434)

- dre:571431

- Down regulated genes

571431(-6.42862549702529)

Close
